# The role of Sequence Type (ST) 131 in adult community-onset non-ESBL-producing *Escherichia coli*bacteraemia

**DOI:** 10.1186/s12879-014-0579-z

**Published:** 2014-11-07

**Authors:** Yi-Hui Wu, Ming-Fang Cheng, Chung-Hsu Lai, Hsi-Hsun Lin, Chih-Hsin Hung, Jiun-Ling Wang

**Affiliations:** Department of Internal Medicine, E-Da Hospital, College of Medicine, I-Shou University, Kaohsiung, Taiwan; Department of Pediatrics, Kaohsiung Veterans General Hospital, Kaohsiung, Taiwan; School of Medicine, National Yang-Ming University, Taipei, Taiwan; Department of Chemical Engineering, Institute of Biotechnology and Chemical Engineering, I-Shou University, Kaohsiung, Taiwan

**Keywords:** E. coli, Bacteraemia, Community onset, Fluoroquinolone resistant, ST (sequence type) 131, Mortality

## Abstract

**Background:**

To compare the epidemiological and clinical features and outcome in clonal group O25b/ST131 and non-clonal group O25b/ST131 in adult patients with non-extended-spectrum B-lactamase (ESBL)-producing *Escherichia coli* (*E. coli*) bacteraemia.

**Methods:**

We collected 371 consecutive isolates with community-onset non-ESBL producing *E. coli* bloodstream infection in 2010 in a 1200-bed hospital in Taiwan. Twenty adult patients with clonal group O25b/ST131 and 40 patients with non-clonal group O25b/ST131 were compared.

**Result:**

Clonal group O25b/ST131 accounted for 5.9% of total isolates. The underlying disease and healthcare-associated risk factors were similar in the case and control groups. Patients with the clonal group O25b/ST131 were less likely to have intra-abdominal infection (0% vs. 22.5%; *p* < 0.05) than patients from the control group. The Day 30 mortality rate was similar in the case and control groups (15% vs. 12.5%).

**Conclusions:**

Clonal group O25b/ST131 was found in both multidrug-resistant and susceptible *E. coli* strains, causing community-onset bloodstream infection. Although O25b/ST131 does not lead to a higher mortality than other isolates, choosing an appropriate antimicrobials in the empirical therapy of community-onset *E. coli* bacteraemia has become more challenging.

**Electronic supplementary material:**

The online version of this article (doi:10.1186/s12879-014-0579-z) contains supplementary material, which is available to authorized users.

## Background

From 2000 to 2006, the *Escherichia coli* clone O25:H4-ST131, which produces CTX-M-15 extended-spectrum B-lactamase (ESBL), was identified in three continents [1]-[3]. ST131's distinctive combination of resistance and virulence, as well as its widespread dissemination among the locals, may underlie its epidemiologic success. Later studies have showed that clone ST131 exists in non-ESBL producing fluoroquinolone-resistant *E. coli* isolates [4]-[8]. ST131 accounted for 25% to 78% of fluoroquinolone resistant *E. coli* infection in the surveillance form in Asia and the US [3]-[8]. In our previous studies, fluoroquinolone (29%) and cefazolin (25%) resistant uropathogens were common in adults with community-onset urinary tract infections (UTI) in a teaching hospital in southern Taiwan. [9] There were several risk factor to be independently associated with community onset UTI fluoroquinolone resistant pathogens (i.e. recent hospitalization, underlying old stroke, and diabetes mellitus) [9]. Although cephalosporin and fluoroquinolone are the recommended antimicrobials to use in community-onset complicated UTI or bloodstream infection, the emergence of clone ST131 with fluoroquinolone resistance in acute pyelonephritis poses a challenge in choosing the adequate antimicrobials. In our institution, we often used 3^rd^ generation cephalosporin such as ceftriaxone or fluoroquinolone such as levofloxacin or ciprofloxacin as empirical antibiotics for community-onset complicated UTI or gram negative bacillary bacteremia.

Our previous studies compared the clinical features and outcome in ST131 vs. non-ST131 in ESBL-producing *E. coli* bacteremia [10]. We found that ST131 was associated with non-catheter related UTI (urinary tract infection) and that it is not associated with higher mortality [10]. According to our review of the literature, we did not find clinical data for clone ST131 in community-onset ESBL-negative *E. coli* bacteremia. To further understand the role of ST131 in community-onset non-ESBL-producing *E. coli* bacteremia, we collected non-duplicated consecutive *E. coli* bloodstream infection isolates in 2010 in a medical center in southern Taiwan. Our goals were to determine the percentage of ST131 in community-onset non-ESBL-producing *E. coli* bloodstream infection and to find out if patients with ST131 clone have clinical features or outcome different from patients with a non-ST131 clone.

## Method

### Study design, setting and participants

**Figure 1:**
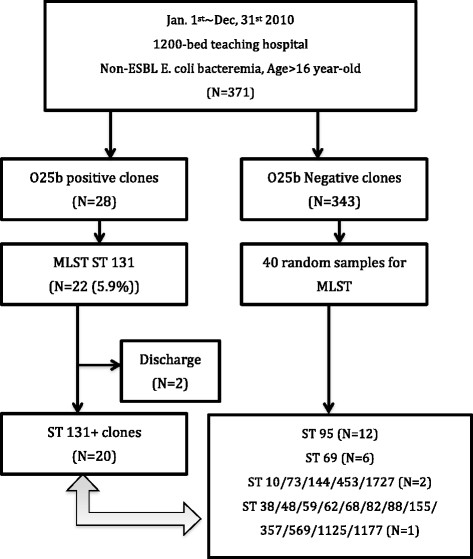
**Algorism of methods and cases analyses.**

The study was done retrospectively and included patients older than 16 years old with at least one positive blood culture of ESBL-negative *E. coli* who were admitted to the E-Da Hospital during a one-year period as Figure 1 (from Jan 1, 2010 to Dec 31, 2010). E-Da Hospital is a 1200-bed major teaching hospital in southern Taiwan that provides both primary and tertiary medical care. Only strains from the first bacteremic episode were included in the analysis. Blood cultures were processed using the automated blood culture system. (BD Phoenix™ Automated Microbiology System)

In the present study, non-susceptibility arbitrarily refers to the category of intermediate and resistant results, obtained using the MIC (minimal inhibitory concentration) value in accordance with the criteria from the Clinical and Laboratory Standards Institute, M100-S21.

### Variables, collection of data and definitions

All patients were evaluated using a standard case recording form. Each clinical course of infection was evaluated and recorded according to the information supplied by primary care physicians and medical records reviewing retrospectively. The diagnosis of infection focus of bacteremia was based on clinical, bacteriological, and radiological investigations.

The following items were recorded for each patient: age; sex; underlying illness; infection syndrome type; history of hospitalization within the previous three months; antimicrobials exposure within the previous three months, existence of a urinary catheter; initial empirical antimicrobial agents; and outcome. If an in vitro active antimicrobial agent was not administered before the final result of blood culture, the procedure was considered a discordant empirical therapy.

This study was supported by the E-Da Hospital Research Committee and approved by the institutional review board of E-Da Hospital. (EDAH IRB No./Protocol No: EMRP-102-048). Because the study was done via medical chart reviewing retrospectively and the pathogens were collected from the stock of the infectious isolates from the laboratory. The IRB waived informed consent requirements due to the research involves no more than minimal risk to the subjects and the waiver will not affect adversely the rights and welfare of the patients.

### Microbiological studies

O25 positve E. coli isolates were determined by the PCR method described by Clermont et al [2]. All O25b positive *E. coli* isolates were characterized by multilocus sequence typing (MLST) using the standard seven housekeeping genes (adk, fumC, gyrB, icd, mdh, purA, and recA), according to the protocol and primers specified by the *E. coli* MLST web site (http://mlst.warwick.ac.uk/mlst/dbs/Ecoli) [2],[11] MLST was also performed on a randomly selected subset of 40 O25b negative E-coli isolates. Clonal relationships were established by pulsed-field gel electrophoresis (PFGE) of XbaI-digested genomic DNA. To compare the band patterns of aggregated data, the GelCompar software package (version 6.0; Applied Maths, Bionumerics) was used. Strains showing 80% or greater banding pattern similarity were considered to have similar or identical electrokaryotypes.

### Statistical analysis

Descriptive statistics, including means, standard deviations, and ranges, were used to analyze continuous variables, whereas percentages and confidence intervals were used to analyze categorical variables. Independent-t test was used for continuous variables. A Chi-square test or Fisher's exact test was used for categorical variables. The variables in bivariate analyses with a *p* value <0.1 were included in a multivariate analysis, which was performed using a logistic regression model to identify factors that independently and significantly affected the outcome. A *p* value <0.05 was considered to be statistically significant. All statistical analyses were done using SPSS 15 for Windows, Chicago, IL, USA.

## Result

Of the 371 non-duplicated consecutive *E. coli* bloodstream infection isolates, 43.9%, 30.7%, 15.4%, 15.4%, 13.2%, and 4.6% were resistant to trimethoprim/sulfamethoxazole (TMP/SMZ), amoxicillin/clavunate, cefazolin, gentamicin, ciprofloxacin, and cefotaxime, respectively (data not shown). The antibiotic resistance of all 371 isolates of clonal group O25b/ST131 and non clonal group O25b/ST131 were shown in the Additional file 1: Table S1. Clonal group 131 were more likely to have TMP/SMZ, ciprofloxacin, cefmetazole, and gentimicin resistance (P < 0.05). Clonal group O25b/ST131 (n = 22) accounted for 5.9% of the total isolates. Two cases of O25-ST131 did not have a complete history in our hospital and were excluded from further clinical analysis.

We collected clinical data from the remaining 20 cases of clonal group O25b/ST131 and 40 randomly selected cases of non-O25b/ST131 genotypes, which were used as controls. The most predominant ST types in the 40 non-O25b/ST131 isolates included ST95 (n = 12) and ST 69 (n = 6). The underlying co-morbidity and healthcare-associated risk factors (such as hospitalization and antimicrobials use) were similar in the case and control groups, as shown in Table 1. Patients with clonal group O25b/ST131 were less likely to have intra-abdominal infection (0% vs. 22.5%; *p* < 0.05) than patients with non-O25b/ST131 clones. Nine cases (15%) received discordant antimicrobials before susceptibility data were available included levofloxacin (n = 4), cephazolin (n = 2), ceftriaxone (n = 1), cefuroxime (n = 1) and amoxicillin/clavunate (n = 1). There were 20% patients in clonal group O25b/ST131 and 12.5% in non-clonal group O25b/ST131 that received discordant antimicrobials before susceptibility data were available (*p* = 0.464).

**Table 1:** **Clinical features and epidemiology data in cases with clonal group O25b/ST131 and control group**

Parameter	Non clonal group O25b/ST131	Clonal group O25b/ST131	P Value
	N = 40	N = 20	
Male sex	10(25)	8(40)	0.232
Age	67.0 + -13.9	66.0+/-17.7	0.807
Underlying disease			
Diabetes mellitus	16(40.0)	6(30)	0.449
Hepatobiliary disease	16(40.0)	8(40)	1.000
Renal structure abnormality	3 (7.5)	4(20)	0.208
Chronic renal disease	4(10)	4(20)	0.422
Liver cirrhosis	8(20)	2(10)	0.471
Malignancy	8(20)	7(35)	0.206
Bedridden	4(10)	2(10)	1.000
Healthcare-associated risk factor			
Hospitalization in previous 6 months	20(50)	6(30)	0.141
Antimicrobials exposure in 3 months			
B-lactam antimicrobials	17(42.5)	6(30)	0.102
Fluoroquinolone	0(0)	2(10)	
Recent operation	5(12.5)	1(5)	0.653
With Foley catheter	5(12.5)	0(0)	0.159
Infection syndrome			
Urinary tract infection	22(55.0)	12(60)	0.713
Intra-abdominal infection	9(22.5)	0(0)	0.023
Outcome			
Discordant antimicrobials	5(12.5)	4(20)	0.464
Shock	6(15)	4(20)	0.718
Length of stay >2 weeks	11(27.5)	6(30)	0.839
Day 30 mortality	5(12.5)	3(15)	1.000

**Figure 2:**
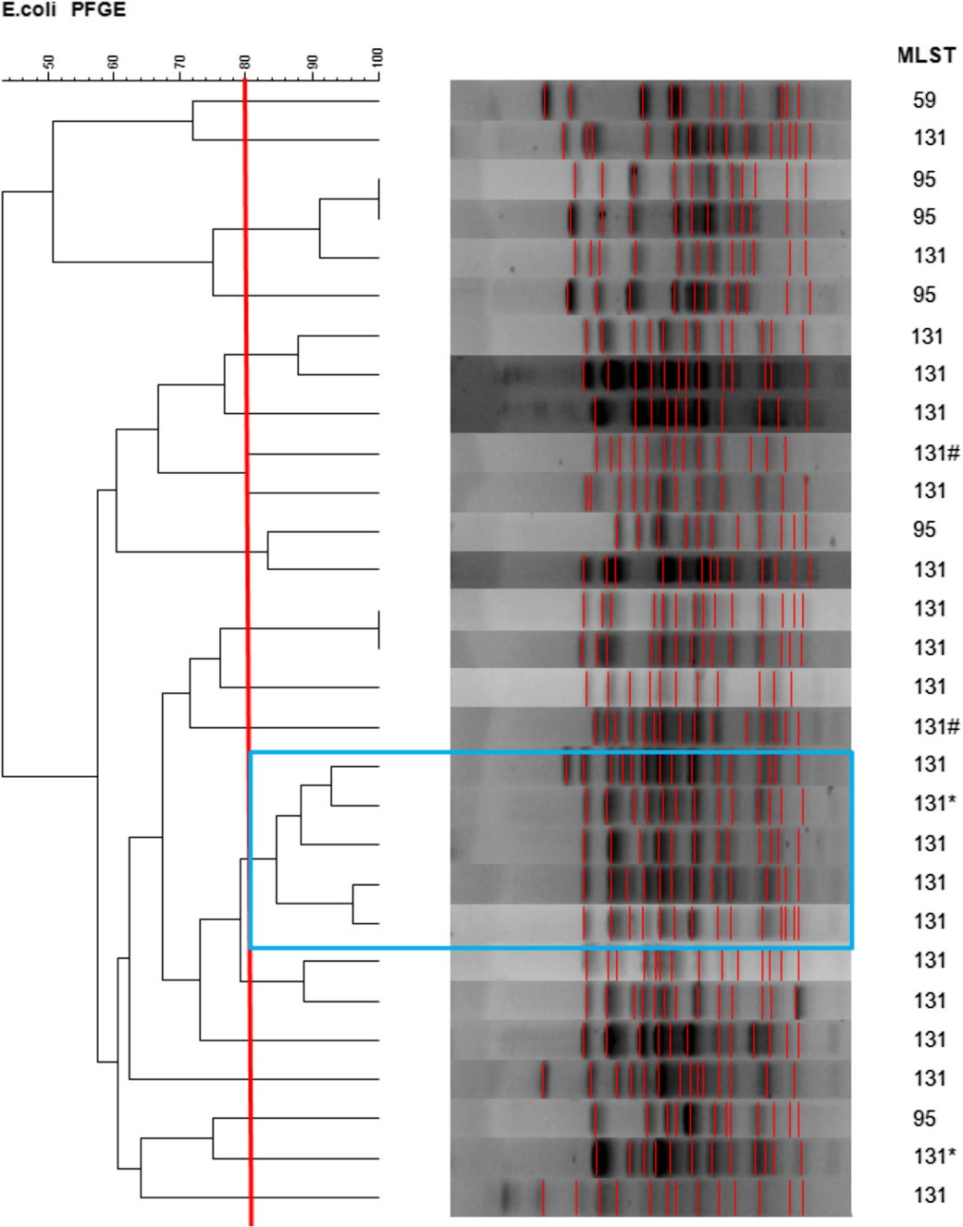
**Pulsed-field gel electrophoresis (PFGE) of XbaI-digested genomic DNA of 23 clonal group O25b/ST131 isolates and five isolates with ST95 and one isolate with ST59.** The major pulsotype was marked. Note: 1. One case with repeated bacteremia (two isolates #) in the same year. 2. Two isolates* were excluded from the clinical analysis due to incomplete clinical data in the medical chart.

The Day 30 mortality rate was similar in the clonal group O25b/ST131 and non-clonal group O25b/ST131 (12.5% vs. 15%, *p* = 1.00), but patients infected with the clonal group O25b/ST131 were more likely to have ESBL *E. coli* infection in the following two years than patients infected with the non-ST131 group (20% vs. 2.5% *p* < 0.05) (data not shown). However, we don't have the following ESBL *E. coli* isolates for further pulsotype study. The univariate risk factors for Day 30 mortality included underlying chronic renal failure (OR: 5.6; 95% CI: 3.0-31.0; *p* = 0.046) and solid organ cancer (OR: 7.0; 95% CI: 1.4 -34.2; *p* = 0.016). The only independent risk factor of Day 30 mortality in multivariate analysis (logistic regression, backward Wald) was chronic renal failure (OR: 16.4; 95% CI: 1.3-212.5; *p* = 0.032) and a primary urinary tract source of infection was associated with lower day 30 mortality (OR: 0.042; 95% CI: 0.002-0.76; *p* = 0.032) (Table 2). Clonal group O25b/ST131 was not related to Day 30 mortality in neither univariate nor multivariate analysis. The resistance percentage of these 60 isolates was shown on Table 3. The PFGE result is shown in Figure 2. There were several pulsotypes in clonal group O25b/ST131, and one major pulsotype included five isolates.

**Table 2:** **Univariate and multivariate analysis in risk factor of Day 30 mortality**

Parameter	Univariate	Multivariate
	Crude OR (95% CI)	Adjusted OR (95% CI)
Chronic renal disease	5.6 (3.0-31.0)	16.4 (1.3-212.5)
Urinary tract infection	0.08 (0.09-0.72)	0.042 (.002-0.76)
Malignancy	7.0 (1.4 -34.2)	Not significant
Clonal group O25b/ST131	1.002 (0.99-1.013)	Not significant

**Table 3:** **Percentage of antibiotic resistance in ST 131 clones and nonST131 clones (N = 60)**

Resistance percentage	O25b-ST131	Non O25b-ST131	P value
(%)	N = 20	N = 40	
Amox/Clavu	6(30)	12(30)	0.788
TMP/SMZ	14(70)	20(50)	0.141
Ciprofloxacin	7(35)	4(10)	0.018
Cefazolin	6(30)	5(12.5)	0.099
Cefmetazole	4(20)	1(2.5)	0.038
Cefotaxime	2(10)	0(0)	0.107
Piperacillin	16(80)	26(65)	0.232
Gentamicin	11(55)	6(15)	0.001

Note: Amox/Clavu denotes as Amoxicillin/Clavunic acid, TMP/SMZ denotes as Trimethoprim/sulfamethoxazole.

No resistance of cefepime, imipenem, amikacin found in these two groups.

## Discussion

We have shown that O25-ST131 is also the important clone in non-ESBL-producing *E. coli* bacteremia. Clonal group O25b/ST131 accounted for 5.9% in total isolates and had a higher proportion in gentamicin, TMP/SMZ and fluoroquinolone resistant isolates. Clonal group O25b/ST131 existed in both multidrug-resistant and susceptible *E. coli* strains causing community-onset bloodstream infection. Most cases with clonal group O25b/ST131 did not have apparent healthcare associated risk factors. This finding answers a previous suggestion that the emerging of clonal group O25b/ST131 may be explained by the enhanced ability to cause extraintestinal infection rather than selection by antimicrobial agents used in hospitals. Current study excluding ESBL positive isolates showed approximately one fourth of fluoroquinolone resistant *E. coli* bacteremia was O25-ST131. Similar to a previous study, although more present in multidrug-resistant isolates, ST131 was found in both antimicrobials susceptible and resistant blood isolates [11],[12]. In a bacteremia study in San Francisco, ST131 accounted for approximately 20% of all drug-resistant and susceptible isolates [11]. Uchida et al. investigated 219 fluoroquinolone-resistant *E. coli* strains in Asian and found that 32% strains were from the serogroup O25 [7]. In Korea, 25% of community-onset urine *E. coli* isolates were ST131 from November 2006 to August 2007 [8].

Similar to a previous study, O25-ST131 was less likely to be associated with intra-abdominal infection and more likely to be related to UTI [5]. In a study from Spain, some healthcare associated risk factors such as diabetes, bedridden status, and antimicrobials exposure were independent risk factors for clone ST131 [13]. A trend for fluoroquinolone exposure in clonal group ST131/O25b was observed in our study but did not reach statistical significance. However, the other correlations were not found in our series, which is possibly explained by the fact that we included only community-onset bacteremia, and patients with some healthcare-associated risk factors may have been excluded. Similar to the study in Spain, mortality was similar between the ST131 and non-ST131 groups [13]. Our study is also in agreement with a previous murine sepsis model in which neither ST131 status nor fluoroquinolone resistance correlated with mortality [14]. More cases of clone ST131 had a higher chance of ESBL *E. coli* infection in the following two years than cases of non-ST131. It remains to be clarified if this phenomenon is related to the colonization and transmission features of ST 131. Nevertheless, a study from France showed that ST131 had high intestine colonization and urinary tract infection abilities, and according to a recent report, ST131 clone was associated with recurrent or persistent urinary tract infection [15],[16].

In our study, despite the high percentage use of discordant antimicrobials in both the ST131 and non-ST131 groups, the mortality was not changed much. Taking into account the predominance of ST131 in *E. coli* bacteremia infection, the selection of adequate empirical antimicrobials becomes difficult. In Asia, the susceptibility rate to fluoroquinolones in *E. coli* was approximately 70% in the urinary isolates from the SMART study [17]. In community-acquired bacteremic acute pyelonephritis, discordant empirical therapy was associated with a worse early clinical response and longer hospital stay than in concordant therapy [18]-[20]. However, choosing broad-spectrum antimicrobials such as carbapenem in this type of community-onset infection raises the concern of antimicrobials selection pressure.

This study is limited by the following factors. First, this is a single center study in Taiwan that included a moderate number of case numbers, and we do not know if the result can be generalized to other parts of the world. Second, we did not collect isolates from colonization or infection after the bacteremia episode. We ignore if the ESBL isolates cultured after infection have the same genotype as the isolates collected during the non-ESBL producing *E. coli* bacteremia. Third, we used the O25b PCR as a screen and confirm by MLST to identify the clonal group O25b/ST131, some of isolates of ST131 but O25b negative may be missed in this screening method. The clinical significance of clone O25b negative ST131 was not known.

## Conclusion

Our preliminary study shows that clone O25b-ST131 isolates emerged as an important cause of non-ESBL-producing *E. coli* bacteremia in Taiwan. Clonal group O25b/ST131 existed in both multidrug-resistant and susceptible *E. coli* strains causing community-onset bloodstream infection. Although O25b/ST131 does not lead to a higher mortality than other isolates, choosing an appropriate antimicrobials in the empirical therapy of community-onset *E. coli* bacteremia has become more challenging due to the appearance of the ST131 clone.

## Authors' contributions

YHW, JLW, CHH carried out the molecular genetic studies and drafted the manuscript. CHL and HHL participated in the design of the study and collected the bacteria isolates. MFC participated in its design and coordination and helped to draft the manuscript. All authors read and approved the final manuscript.

## Additional file

## Electronic supplementary material

Additional file 1: Table S1.: Percentage of antibiotic resistance in ST 131 clones and non ST131 clones in all isolates (N=371). (DOC 35 KB)

Below are the links to the authors’ original submitted files for images.

Authors’ original file for figure 1

Authors’ original file for figure 2
