# Supplementary material for: The role of Sequence Type (ST) 131 in adult community-onset non-ESBL-producing Escherichia colibacteraemia
Source: BMC Infect Dis. 2014 Nov 7;14:579. doi: 10.1186/s12879-014-0579-z (PMC4234847; doi:10.1186/s12879-014-0579-z)
Supplement: Supplementary file 1 — Additional file 1: Table S1.: Percentage of antibiotic resistance in ST 131 clones and non ST131 clones in all isolates (N=371). (DOC 35 KB) [file 12879_2014_579_MOESM1_ESM.doc]

Additional file 1: Table S1. Percentage of antibiotic resistance in ST 131 clones and non ST131 clones in all isolates. (N=371)

| Resistance percentage (%) | O25b-ST131  N=22 | Non O25b-ST131  N=349 | P value |
| --- | --- | --- | --- |
| Amox/Clavu | 7(31.8) | 107(30.7) | 1.000 |
| TMP/SMZ | 15(68.2) | 148(42.4) | 0.018 |
| Ciprofloxacin | 8(36.4) | 41(11.7) | 0.001 |
| Cefazolin | 6(27.3) | 51(14.6) | 0.110 |
| Cefmetazole | 4(18.2) | 23(6.6) | 0.042 |
| Cefotaxime | 2(9.1) | 15(4.3) | 0.297 |
| Piperacillin | 18(81.8) | 216(61.9) | 0.06 |
| Gentamicin | 13(59.1) | 44(12.6) | <0.001 |

Note: Note: Amox/Clavu denotes as Amoxicillin/Clavunic acid, TMP/SMZ denotes as Trimethoprim/sulfamethoxazole

No resistance of cefepime, imipenem, amikacin found in these two groups.
