# Supplementary figures and images for: The role of Sequence Type (ST) 131 in adult community-onset non-ESBL-producing Escherichia colibacteraemia
Source: BMC Infect Dis. 2014 Nov 7;14:579. doi: 10.1186/s12879-014-0579-z (PMC4234847; doi:10.1186/s12879-014-0579-z)

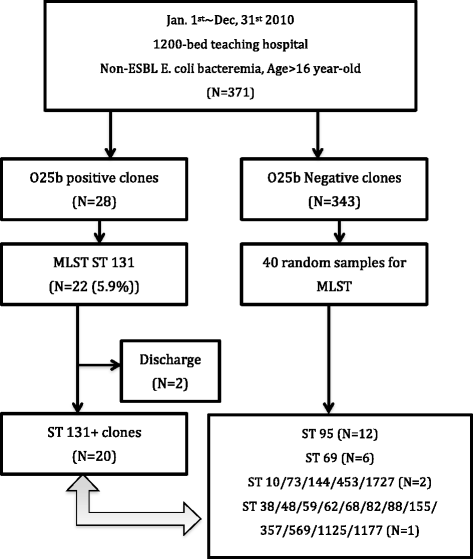

Supplement: Supplementary file 2 — Authors’ original file for figure 1 [file 12879_2014_579_MOESM2_ESM.gif]

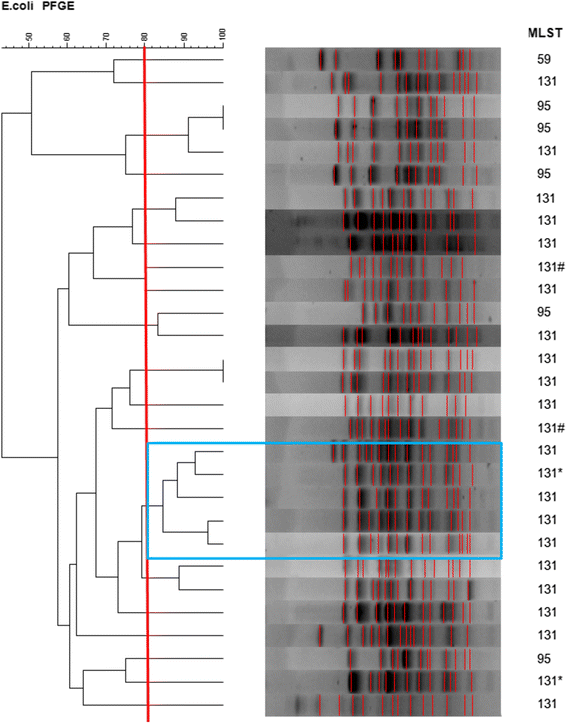

Supplement: Supplementary file 3 — Authors’ original file for figure 2 [file 12879_2014_579_MOESM3_ESM.gif]
